# Integrative Bioinformatics Analysis of Genomic and Proteomic Approaches to Understand the Transcriptional Regulatory Program in Coronary Artery Disease Pathways

**DOI:** 10.1371/journal.pone.0057193

**Published:** 2013-02-28

**Authors:** Rajani Kanth Vangala, Vandana Ravindran, Madan Ghatge, Jayashree Shanker, Prathima Arvind, Hima Bindu, Meghala Shekar, Veena S. Rao

**Affiliations:** 1 Tata Proteomics and Coagulation Department, Thrombosis Research Institute, Bangalore, Narayana Hrudayalaya Hospital, Bangalore, Karnataka, India; 2 Elizabeth and Emmanuel Kaye Bioinformatics and Biostatistics Department, Thrombosis Research Institute, Bangalore, Narayana Hrudayalaya Hospital, Bangalore, Karnataka, India; 3 Mary & Garry Weston Functional Genomics Unit. Thrombosis Research Institute, Bangalore, Narayana Hrudayalaya Hospital, Bangalore, Karnataka, India; Wageningen UR Livestock Research, The Netherlands

## Abstract

Patients with cardiovascular disease show a panel of differentially regulated serum biomarkers indicative of modulation of several pathways from disease onset to progression. Few of these biomarkers have been proposed for multimarker risk prediction methods. However, the underlying mechanism of the expression changes and modulation of the pathways is not yet addressed in entirety. Our present work focuses on understanding the regulatory mechanisms at transcriptional level by identifying the core and specific transcription factors that regulate the coronary artery disease associated pathways. Using the principles of systems biology we integrated the genomics and proteomics data with computational tools. We selected biomarkers from 7 different pathways based on their association with the disease and assayed 24 biomarkers along with gene expression studies and built network modules which are highly regulated by 5 core regulators PPARG, EGR1, ETV1, KLF7 and ESRRA. These network modules in turn comprise of biomarkers from different pathways showing that the core regulatory transcription factors may work together in differential regulation of several pathways potentially leading to the disease. This kind of analysis can enhance the elucidation of mechanisms in the disease and give better strategies of developing multimarker module based risk predictions.

## Introduction

Deregulation of transcription program leads to important changes during onset and progression of several diseases. The risk of developing cardiovascular diseases is signified by modulation of several pathways like inflammation, coagulation, obesity, diabetes, renal function, stress and oxidative stress measured by respective biomarkers representing each pathway. Thus integrating the transcriptional changes which lead to differential expression of biomarkers and pathophysiology are needed to identify the best discerning molecular biomarkers. One of the most important aspects of translational research is the need to discover novel cardiovascular disease biomarkers for early detection of the pathogenesis, inform prognosis, guide therapy and monitor the disease progression. Despite several expectations from ‘omics technologies, elucidation of accurate and discriminating disease biomarkers for the clinical management still remains a challenge. Many studies have focused on using microarray and proteomics technologies for novel biomarker discovery. However, compared with massive knowledge about transcriptome/proteome, we have surprisingly little knowledge about regulatory mechanisms underlying the biomarker diversity. To analyze the transcriptional regulatory programs, gene expression, proteomics, and integrative computational approaches that integrate regulatory sequence data are needed. So far many approaches have been developed in lower organisms like yeast to correlate between the presence of cis-regulatory motifs and expression values [1], [2]. On the other hand, such important analyses in higher organisms like humans are just starting [3], [4].

In this study we used transcription factor profiles, gene expression and proteomic expression data in combination with bioinformatics analysis to identify the core transcription factors which might regulate several interactive pathways associated with coronary artery disease (CAD). In our approach, we selected candidate biomarkers representatives of CAD associated pathways whose promoter regions were analyzed. Gene expression studies were carried to identify the expression levels of the transcription factors (TFs) which regulate the biomarkers and have correlated them with proteomic expression of biomarkers. Using this approach we have dissected a core set of transcriptional regulatory program which might give a better understanding of the association of differential expression of biomarkers/pathways with CAD.

## Methods

### Selection of Biomarkers

Guided by recent reviews and research articles on biomarkers in cardiovascular diseases, a list of 31 known biomarkers were compiled from 7 different pathways shown to be highly associated with CAD. Biomarker selection was based on the association of individual biomarkers to CAD and their ability for risk prediction. Inflammation [5]–[12] and coagulation are the two major pathways known to be associated with CAD and therefore majority of biomarkers were selected from these pathways (inflammation: Interleukin 6 (IL6), Interleukin 8 (IL8), Interleukin 10 (IL10), Interleukin 12A (IL12A) [12], Interleukin 12B (IL12B) [12], Interleukin 18 (IL18), Monocyte chemoattractant protein-1 (MCP-1 or CCL2), High sensitive C reactive protein (CRP), Interferon gamma (IFNG), Matrix metalloprotease-9 (MMP9) and secretary Phospholipase A2 (sPLA2 or PLA2G2A) and Gamma-glutamyltransferase 5 (GGT5) [7], Coagulation [8]–[13]: Factor VII, Fibrinogen alpha, beta, gamma, Prothrombin, Plasminogen activator inhibitor-1, Plasminogen (PLAT), Tissue factor [14], von Willebrand Factor, Platelet derived growth factor (PDGF) [15].

The other biomarkers selected were from pathways like cell adhesion (Clusterin or CLU and P-selectin or SELP) [16]–[18], obesity (Adiponectin or ADIPOQ and Leptin or LEP) [19], [20], oxidative stress (Myeloperoxidase or MPO) [9]–[12], Stress (HSP27 or HSPB1, HSP60 or HSPA1A, HSP70 or HSPD1) [21] and renal function marker (Cystatin C or CST3) [22].

### Study Population

Two independent subsets were selected from the Indian Atherosclerosis Research Study (IARS), which is an ongoing, family based epidemiological study initiated in 2003 to investigate the genetic, conventional and environmental factors associated with CAD in Asian Indians living in the Indian subcontinent [23]. For the microarray studies, whole blood samples of 10 CAD affected subjects and 10 unaffected controls were selected. Similarly serum samples of 413 CAD affected subjects and 413 unaffected family members were selected from IARS population for performing biomarker assays. For both sets of samples affected and unaffected were matched with respect to age and gender.

Novel biomarker discovery is a specific aim of this study. For this study, families were enrolled from two Indian cities: Bangalore and Mumbai. Subjects were recruited through a proband with i) angiographic evidence of CAD (males ≤60 years and females ≤65 years at onset), ii) a family history of CAD/CVD and iii) undergoing therapeutic/surgical treatment at participating hospitals. Extended family members both affected and unaffected were enrolled provided they met the recruitment age of 18 or above. Blood sampling and physical examinations were conducted and subjects with cancer, cardiomyopathy, rheumatic heart disease, liver or renal disease and concomitant infection were excluded. Prevalence of diabetes and hypertension in study participants was ascertained based on self-report, use of prescription medications and medical records of therapeutics. The information from medical records was obtained by trained clinical research assistants under the guidance of a physician, following a standardized protocol. Follow-up of the subjects began in 2005 by telephone and continues to date. The IARS study has been designed on the guidelines of the Indian Council of Medical Research for studies on human subjects and is approved by the Thrombosis Research Institute ethics committee [23]. All participants gave their written informed consent to participate in the study.

### Gene Expression Studies

The gene expression studies using microarray experiments was independently performed and specific TFs and biomarker expression data was utilized for further analysis (10 healthy controls and 10 cases from the IARS cohort). Total RNA was extracted from each sample using QIAamp RNA Blood mini kit (Qiagen) techniques. In brief, 3 ml of fresh blood was collected in EDTA coated tubes. 1.5 ml of that was taken for RNA isolation. Manufacturer recommended protocol and reagents were used. After isolation, RNA was cleaned up using RNeasy mini elute cleanup kit (Qiagen). The RNA quantity and quality were determined by NanoDrop ND-1000 UV-VIS Spectrophotometer and Agilent Bioanalyzer 2100 (Agilent Technologies). The study design was formulated using one color microarray for cy3 using 8×60k slides from Agilent technologies.

#### Sample preparation

One color spike mix was prepared and 2 ul of this mix was added to 50 ng (1.5 ul) of each RNA sample individually. RNA was reverse transcribed to cDNA using high capacity cDNA reverse transcription kit (Applied Biosystems, USA). cDNA was then converted into cRNA, with simultaneous dye labeling. The labeled/amplified RNA samples were purified using Qiagen RNeasy mini kit as per the manufacturer’s protocol. The purified RNA (1.0 to 2.0 ul) was quantified using NanoDrop ND-1000 UV-VIS Spectrophotometer. Hybridization was performed as per the manufacturer’s protocol.

#### Hybridization

50 ul of blocking agent was added to 5 ug of cRNA for preparation of hybridization samples followed by 10 ul of fragmentation buffer and the final volume was adjusted to 250 ul. To this 250 ul of hybridization buffer was added and mixed thoroughly. This mix was slowly dispensed on to the gasket well in a drag and dispense manner. Array was slowly placed on the “active side” down on SureHyb gasket slide, covered with the SureHyb chamber cover and assembled using clamp. The hybridization was carried out at 65°c for 17 hours in a hybridization oven.

#### Scanning

The slides were washed with buffer1 for 1 minute; pre warmed buffer2 for 1 minute and dried for 10 seconds. The slide was placed in slide holder and scanned using microarray scanner. Upon completion the data generated was taken for further processing.

The microarray data analysis was carried out using ‘R’ and bioconductor packages. Normalization of raw data was done on LIMMA (Linear Model for micro array data), a package for the analysis of microarray data, for the assessment of differentially expression. Between arrays normalization was performed by Quantile method. The normalized data was taken for further analysis and the difference in expression between the cases and controls were done using T-test.

### Biomarker Assays

24 biomarkers were screened using ELISA, Cytometric bead array assays and automated coagulation analyzer (ACL300) in 816 subjects (413 cases and 413 matched controls). Affected and unaffected subjects were selected from the Indian Atherosclerosis Research Study (IARS) cohort.

Biomarkers IL6, MCP-1,MMP9, P-selectin, PDGF, PAI-1, Tissue Factor or Coagulation factor 3, vWF, Adiponectin, Leptin and Cystatin C were obtained from R&D Systems, Minneapolis, USA. GGT5 expression kit was from USCN Life Sciences, Houston, USA, sPLA2 from Cyman Corporation, USA, Clusterin from BioVendor Laboratory medicine Inc, Modrice, Czech Republic, MPO levels were measured using kits from Mercodia (Uppsala, Sweden), and CRP levels were measures using Roche latex Tina quant kit (Roche Diagnostics, Switzerland). Stress markers Hsp60, HSP27 andHSP70 were assayed using Stressgen Bioreagents, Victoria, Canada. The ELISA plates were read on a plate spectrophotometer (PowerWave™ XS, Bio-Tek® Instruments, Inc., Vermont, USA). The fold change for each biomarker was calculated.

2 biomarkers Interleukin 10 (IL-10) and Interferon gamma (IFNG) were assayed by Cytometric bead array assay (CBA) following manufacturer’s instruction. The coagulation markers namely plasma fibrinogen and Factor VII and Prothrombin were measured by using clotting assay on automated coagulation analyzer (ACL 300, Instrumentation Laboratories, Milano, Italy).

### Bioinformatics Analysis

**Figure 1:**
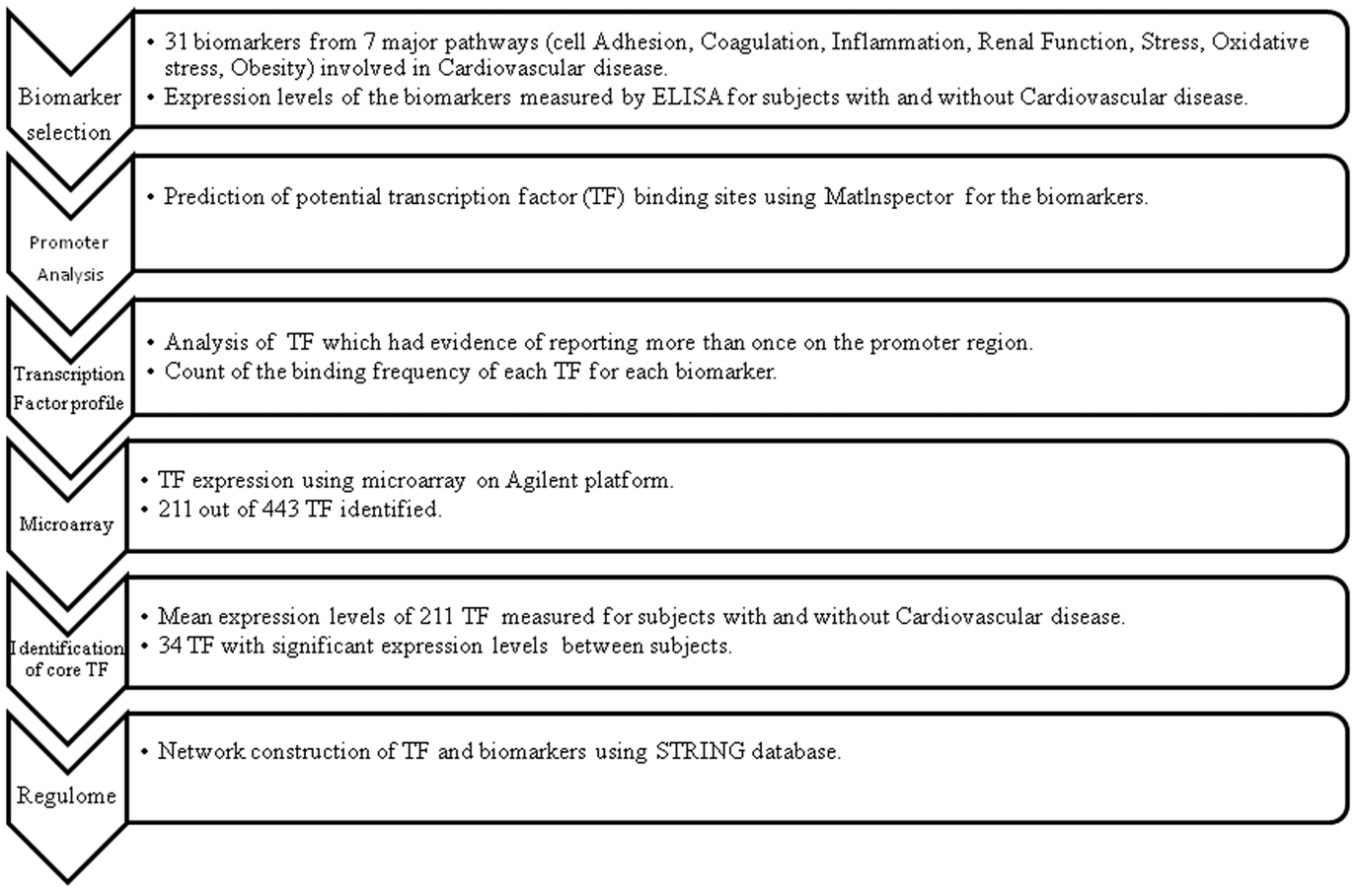
Methodology for regulome and network module analysis.

As shown in the figure 1, promoter analysis to find the core TFs for the 31 biomarkers belonging to different pathways was carried out on MatInspector [24] program from Genomatix software suite. The TFs which had experimental evidence of more than one were chosen for further analysis. The number of binding sites for each TF was noted and the total TF binding sites for each TF family were calculated. This was considered proportional to the number of times the TF binds to the DNA. An entire profile of the significant TF binding pattern was generated and was represented by a heat map using Matrix2png [25]. Each TF was assigned to the family they belong and the common TFs regulating the different pathways were identified using VENNY [26].The network between the significant TFs and the biomarkers was built on STRING [27].

## Results and Discussion

### Identification of Common Transcription Factors Regulating CAD Pathways

**Figure 2:**
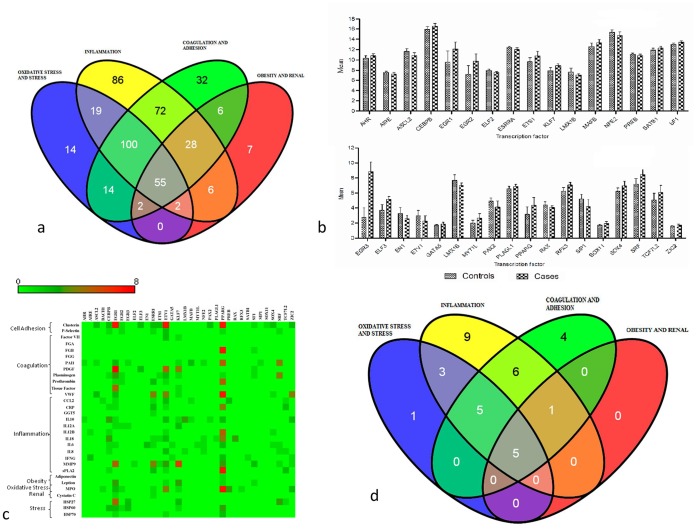
Identification of core transcription factors. a. Venn-diagram of the 443 Transcription factors regulating the pathways in CVD and identification of 55 core transcription factors. b. Mean expression levels of significant transcription factors obtained from microarray between cases and controls. c. Number of binding sites of 34 expressed transcription factors in the biomarkers from 7 different pathways. d. Venn-diagram of the 34 significant Transcription factors regulating the pathways in CVD and identification of 5 core transcription factors as PPARG, EGR-1, ETV-1, KLF-7, and ESRRA.

The 31 biomarkers selected were belonging to seven different pathways representing the pathological progression of the disease. The promoter regions of these 31 biomarkers were analyzed for TF binding sites using Genomatix software. 443 TFs were identified to be binding to the biomarker promoter regions of which 55 were common for all the 31 biomarkers (figure 2a). These 55 TFs could potentially regulate different pathways like stress, oxidative stress, inflammation, coagulation, cell adhesion, obesity and renal function. Furthermore we found that these 443 TFs belong to 124 different families and the core 55 (Table S1) were representatives of 23 TF families.

### Transcription Factor Expression Modulation in CAD Associated Pathways

The 443 TFs identified were predicted using Genomatix software. However, it is important to know how many of these predicted TFs were really expressed in the blood samples of CAD affected subjects and controls. Therefore microarray gene expression data was used and was found that only 34 TFs were actually differentially expressed with a significant p-values (p value >0.05) (Table S2) (figure 2b). The differential expression of these TFs might lead to influence on the expression pattern of the biomarkers, thus modifying the pathways.

### Core Transcription Factors and Regulatory Influence on Pathways

The number of binding sites for individual TFs in each of the biomarker promoters may influence the level of the expression of the biomarkers. Therefore, we analyzed the number of the binding sites of 34 expressed TFs in the promoter sequences of the biomarkers (figure 2c and Table S3). 5 TFs were found to be binding to all the biomarkers in 7 different pathways (figure 2d).

Of the 34 TFs which were found to be expressed in the blood tissue of CAD affected patients and control subjects, the 5 major TFs which seem to regulate the majority of biomarkers associated with CAD have been identified as PPARG, EGR1, ETV1, KLF-7 and ESRRA.

We found that Peroxisome proliferator-activated receptor gamma (PPARG) has the highest number of binding sites in majority of the biomarkers with 76 binding sites in total. PPARG seems to regulate cell adhesion (7 binding sites in promoter of Clusterin and 1 in p-selectin), coagulation (7 binding sites in fibrinogen B, 5 in PAI1, 1 in plasminogen, 5 in prothrombin, 7 in vWF), inflammation (CRP 4 binding sites, IL10 2 binding sites, 5 in IL12B, 4 in IL18, 2 in IL6, 2 in interferon gamma, 3 in MMP9, and 7 in sPLA2), obesity (1 each binding sites in Adiponectin and Leptin), oxidative stress (7 binding sites in Myeloperoxidase) and stress (3 binding sites in HSP60 and 2 in HSP70).

Early growth response 1 (EGR1) is the second TF which binds to majority of biomarker promoters with a total of 52 binding sites. It binds to cell adhesion protein clusterin’s promoter at 7 sites, 21 in coagulation (2 binding sites in PAI1 promoter, 8 in PDGF, 3 in plasminogen, 1 in prothrombin, 5 in tissue factor, and 2 in vWF), 10 binding sites in promoters of inflammation pathway genes (1 in CCL2, 1 in CRP, 1 in IL10, 1 in IL12A, 1 in IL6, 5 in MMP9), 3 binding sites in the promoter of leptin the obesity marker, 2 in MPO promoter which is a oxidative stress marker, 1 in Cystatin-C promoter the renal function marker and 8 in stress related biomarkers (5 in HSP27, 2 in HSP60, 1 in HSP70 promoters).

ETS translocation variant 1 (ETV1) which is ETS family member has 43 binding sites in different biomarkers representative of different pathways. The ETS family members regulate many important functions like cell growth, angiogenesis, migration, proliferation and differentiation. ETV1 has 9 binding sites in promoters of cell adhesion biomarkers (7 in Clusterin, 2 in P-selectin), 14 binding sites in coagulation proteins (2 in Factor VII, 5 in PDGF, 2 in plasminogen, 1 in prothrombinand 4 in vWF), 14 binding sites in promoters of inflammatory markers (2 in CCL2, 1 in CRP, 2 in IL10, 1 in IL12B, 2 in IL18, 2 in IL8, 3 in MMP9 and 1 in sPLA2), 1 binding site in the promoter of the obesity marker Leptin, and 4 in the promoter of Myeloperoxidase biomarker for oxidative stress.

The next important TF, Kruppel-like factor 7 (KLF-7) belongs to Kruppel-like factors family which are zinc finger proteins binding to CACCC motif and also to SP1 binding sites with a total of 25 promoter binding sites in different biomarkers. KLF-7 does not seem to regulate both the biomarkers of cell adhesion but it has 9 binding sites in the promoters of coagulation pathway biomarkers (1 in Factor VII, 1 in PAI1, 4 in PDGF, 1 in Plasminogen, 1 in Tissue factor, and 1 in vWF), 9 binding sites in the promoters of inflammation pathway biomarkers (1 in IL10, 2 in IL12A, 6 in MMP9), 3 binding sites in promoters of Leptin the biomarker for obesity, 1 in MPO marker for oxidative stress and 3 in stress markers promoters (2 in HSP27, 1 in HSP60).

23 binding sites were observed for Estrogen-related nuclear receptor alpha (ESRRA) TF, which is a zinc finger protein regulating several pathways like cell proliferation, differentiation, transcription and receptor functions. ESRRA has 3 binding sites in the promoters of cell adhesion (2 in clusterin and 1 in P-selectin), 6 binding sites in the promoters of two biomarkers involved in coagulation (2 in prothrombin and 4 in vWF), 12 binding sites in inflammatory biomarkers (1 in CCL2, 2 in CRP, 1 in GGT5, 1 in IL18, 1 in IL6, 4 in MMP9, and 2 in sPLA2), one binding site present in MPO the oxidative stress marker and Cystatin-C the renal function marker.

The rest of the 29 TFs are represented in low number of binding sites in the promoters of the biomarkers evaluated by us. However, CEBPB (the CCAAT binding transcription factor beta) and serum response factor (SRF) also seem to have good number of binding sites with 17 and 15 respectively in the 31 biomarkers.

### Combinatorial Regulation of CAD Pathways

Apart from above mentioned TFs being highly represented, we found that there are other combinations of TFs regulating the 7 pathways (figure 2d). There were 4 biomarkers used in this study belonging to oxidative stress (MPO) and stress (HSP27, HSP60 and HSP70) and apart from the 5 core transcription factors, we found that only one TF Regulatory factor X 3 (RFX3) is a specific regulator of MPO. 3 TFs TCF7L2, BACH1 belonging to cap'n'collar type of basic region leucine zipper factor family (CNC-bZip) and Prolactin regulatory element-binding protein (PREB) are co-regulators of oxidative stress, stress and inflammatory pathways biomarkers. Similarly 5 TFs EGR2 CEBPB, EGR3, SRF and ZIC2 combinations regulate the biomarkers of oxidative stress, inflammation, coagulation and cell adhesion.

Inflammation pathway biomarkers we specifically regulated by 9 TFs namely AIRE, ELF2, EN1, GATA5, LMX1B, MYT1L, PLAGL1, RAX, and SATB1. 6 TFs AHR, ETS1, MAFB, NFE2, PAX2 and SOX4 were found to be potential regulators of inflammation, coagulation and adhesion pathway biomarkers. Similarly 4 TFs ASCL2, ELF3, SOX11 and SIP1 were found to specifically regulate the coagulation and cell adhesion biomarkers. SF1 TF was found to be having potential binding sites in biomarkers from different pathways like cell adhesion, coagulation, inflammation and obesity.

### Differential Expression of mRNA and Protein of Biomarkers

**Figure 3:**
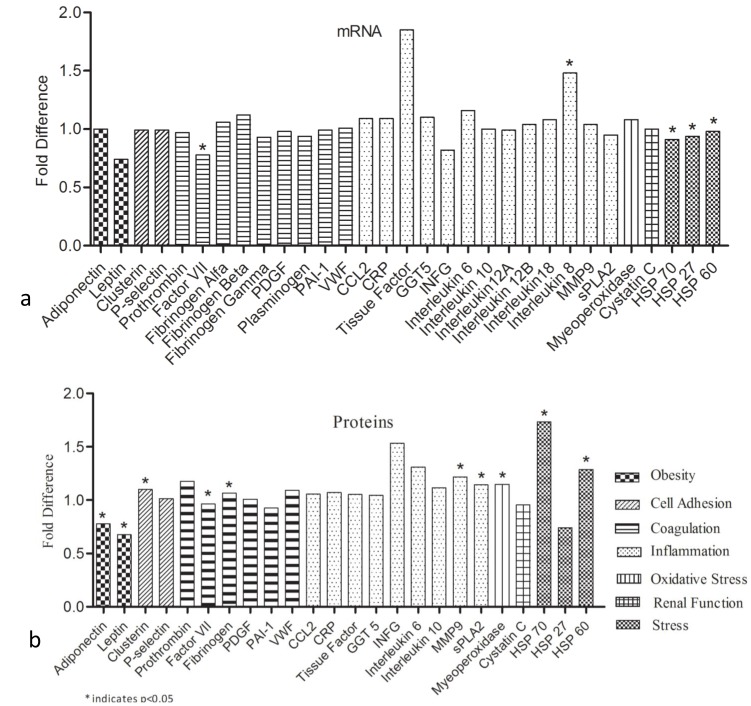
Modulation of m-RNA and protein expression profiles. a. Fold change in the mRNA expression of biomarkers from microarray data. b: Fold change in the expression of biomarkers at protein level.

The mRNA expression in 20 subjects (10 affected and 10 unaffected subjects) and protein expression levels in 816 subjects (408 affected and 408 unaffected subjects) of 7 pathways representative biomarkers were performed. The mRNA expression levels of the 24 biomarkers (fibrinogen isoforms, alpha, beta and gamma were evaluated individually) were taken from the microarray experiments (figure 3a). The data suggests that 5 biomarkers (Factor VII, IL8, HSP70, HSP60 and HSP27) were significantly differentially expressed at the mRNA level. Furthermore, we assayed 24 biomarker proteins (whole fibrinogen was evaluated in the protein study) (figure 3b) and found that Adiponectin, Leptin, Clusterin, Factor VII, Fibrinogen, MMP9, sPLA2, Myeloperoxidase, HSP70 and HSP60 were significantly differentially expressed.

### Network of Biomarkers and Transcription Factors

**Figure 4:**
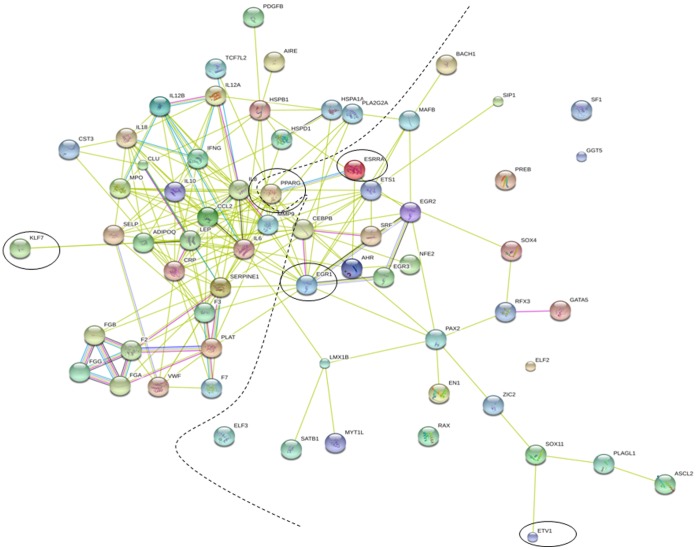
Transcription factor and Protein network. Circled Transcription factors are common among the pathways. The dashed line represents the demarcation between the Transcription factor regulation in nucleus and biomarker expression in extracellular matrix.

Based on the transcription factors identified and biomarkers analyzed we used STRING database to develop a network model (figure 4). As seen in the figure 4, the TFs PPARG, EGR1, ESRRA, CEBPB, ETS1, LMX1B and MAFB are the direct networking members with the biomarkers. Of these 7 TFs, PPARG and EGR1 are highly networked and are interfacing with the biomarkers. PPARG seems to be associated with other transcription factors like ESRRA, AHR, EGR1, TCF7L2, and CEBPB, potentially co-regulating the target biomarkers of the TFs. SimilarlyEGR1, is associated with SRF, EGR3, EGR2, PAX2, CEBPB, and MAFB transcription factors. These kinds of networks suggest the collaborative interactions between several TFs in regulating the biomarkers.

### Interaction between Pathways as Modules

As seen in the figure 4, the biomarkers also are highly networked and functional associations are clearer in the network. For example, the early phase of atherosclerosis involves the recruitment of inflammatory cells from the circulation and their transendothelial migration. This process is predominantly mediated by cellular adhesion molecules, which are expressed on the vascular endothelium and on circulating leukocytes in response to several inflammatory stimuli. In our study the cell adhesion molecules Clusterin and P-selectin were similarly expressed in our data (figure 3b) could be regulated together by core TFs PPARG, EGR1, ETV1 and ESRR1 (figure 2c). However, Clusterin associates with other biomarkers like MPO (oxidative stress), HSP27 (HSPB1, Stress), PAI1 (SERPENE1, coagulation), Leptin (obesity) which represent markers from different pathways (figure 4). Similarly P-selectin shown to be associated with MPO (oxidative stress), members of inflammation like IL6, CCL2, IFNG, IL8, IL10, coagulation members like vWF and Factor 3.These kind of networks form a module consisting of several markers from different pathways and differential expression of these modules might be a better way to look at the functional association of pathways.

Another set of biomarkers forming a novel module of network biomarkers are Factor 3 and vWF. These two biomarkers form a good network with biomarkers (figure 4) of other pathways like inflammation (IL6, CRP, IL8, CCL2, IL10), obesity (ADIPOQ, Leptin), cell adhesion (P-selectin) and other coagulation members (PAI1, F7, vWF FG alpha and beta). Most of the coagulation biomarkers seem to be regulated by EGR and ETS family TFs. In the inflammation pathway, IL6 seems to be networked with several of other pathways like oxidative stress (MPO), coagulation (PAI1 or SERPENE1, Factor 3, Fibrinogen beta), stress (HSP27 or HSPB1, HSPD1), cell adhesion (P-selectin) and obesity (Adiponectin and Leptin). Similarly is the case with other inflammatory markers like hsCRP, MMP9, IL8, and others.

### Transcription Regulation and Identification of Core Regulators of CAD

Our present study shows how the regulatory mechanisms might play a major role in differential expression of biomarkers associated with CAD and how the functional modules are formed from different CAD associated biomarkers. Using an integrative strategy of unifying the genomic and proteomic data by bioinformatics tools, we have identified a set of transcription factors which form the core regulators and also pathway specific regulators. Using the literature survey we have identified key biomarkers which are representatives of molecular pathways shown to be associated with CAD independently. Using bioinformatics tools we have identified core regulatory elements which might play an important role in regulating the differential expression of these biomarkers (figure 2a). However, it is important to know how many of these predicted transcription factors are really expressed and using the microarray experiments we have shown that 34 out of 443 TFs are differentially expressed (figure 2b). The differential expression patterns were not drastic however similar to other studies the individual TFs even though weakly differentially expressed can play an important role in elucidating the underlying biology of the disease. The genes with significant individual discriminatory capacity tend to represent “passenger” genes, whereas “driving” disease networks can include combinations of relatively weakly differentiating genes [28], [29]. Deregulation of transcriptional program leads to development and progression of several diseases and many TFs have been identified as potential biomarkers [30]. In this study we devised a strategy of deciphering transcriptional programs from microarray data and have shown that the core regulators are differentially expressed in cases versus controls (figure 2b). This data suggests that at the transcriptional regulatory program in patients affected with CAD were altered. Furthermore, the core TFs PPARG, EGR1, ETV1, ESRRA and KLF-7 being differentially expressed may mean that a combination of these TFs might play a major role in development of CAD. Earlier reports also suggest that another transcription factor GATA2 expression levels and single nucleotide polymorphisms (SNPs) may be associated with family early onset of CAD [31]. Similarly PPARG is shown to play a major role in development of CAD [32] and also specific SNPs in the gene and the promoter binding site of PPARG are shown to be associated with type-2 diabetes, a major risk factor for CAD [33]. In our data PPARG is a major regulator of majority of pathways (figure 2c) and might play a very important role in modulating all the pathways associated with CAD.

EGR-1 has also being shown to be an important member in regulating atherogenesis and seems to co-localize with fibroblast growth factor-2 [34] to endothelial cell microvascular channels in human microvascular channels. It has also been shown that EGR-1 plays a major role in regulating oxidative stress pathway in the disease progression [35] and in our data we observed that myeloperoxidase a marker for oxidative stress is highly regulated (figure 2c) by EGR-1.

The other TFs like ETV-1 a member of ETS transcription factor are needed for normal coronary and myocardial development [36] and ESRRA plays a important role in early development of atherosclerosis [37]. Krüppel-like factors are members of the zinc finger family of transcription factors that have been implicated as playing key roles in regulating cellular differentiation and tissue development. Studies over the past several years support an important role for this family of factors KLF-1 to KLF-6 in endothelial biology [38]. However, KLF-7 is shown to play a major role in olfactory bulb dopaminergic neuron development [39] and its role in cardiovascular disease needs to be understood more.

Unique transcription factors like RFX3 seem to be up regulated in patients with CAD and its binding sites found only on the promoter of MPO the oxidative stress marker. This suggests that a coordinated interaction between the 5 core TFs and RFX3 might be needed to modulate the expression of MPO. Similarly the 9 TF which have binding sites in majority of inflammation pathway biomarkers may play important role along with the core regulators. The specific role of these unique TFs has to be studied further to know potential implications of their differential expression in CAD affected subjects.

### Differential Regulation and Biomarker Network Driven CAD-risk Modules

It is a well know fact that a complex diseases have genes which interact and work cooperatively but till date how they associate with diseases is not completely understood. The interactive network of TFs (figure 4) and the multiple binding sites for these TFs in different pathway representative biomarker promoters suggests that the regulatory networks work together collaboratively. These collaborative regulome may thus lead to important expression changes of biomarkers (figure 3a and b) in turn associating with CAD. The biomarker expression and interaction is needed as the next step to regulation for onset of the disease. These interactomes (figure 4) of biomarkers might work together in specific modular architecture and in our data we see that cell adhesion pathway molecules (clusterin and P-selectin) form a cluster with biomarkers from oxidative stress (MPO), stress (HSP27), coagulation (PAI1) and obesity (leptin) based on the nodes joining these molecules (figure 4). These biomarkers from different pathways may be working in coordination with each other in the early phase of the disease thus forming the risk module for CAD. Similar modular architecture can be found with IL6 associating with oxidative stress (MPO), coagulation (PAI1 or SERPENE1, Factor 3, Fibrinogen beta, stress (HSP27 or HSPB1, HSPD1), cell adhesion (P-selectin) and obesity (Adiponectin and Leptin). Recent published studies also suggest that similar risk modules can exist and interact with neighbors in a collaborative way leading to dysfunction of series of biological processes [40]. In our study the risk modules have biomarkers from different pathways and are not limited to specific pathways. The relationships between the modules might be more with respect to disease but may not with specific pathways to which the biomarkers belong. Therefore our data suggests that biomarkers from different pathways are differentially regulated by combination of core and specific TFs and their interaction may lead to differential expression in the disease condition. Also as seen in our data the disease genes associate through a prescribed communication protocols, like regulome, expression and interactome in shifting the equilibrium in CAD.

## Supporting Information

Table S1 **55 predicted core transcription factors belonging to 23 families.**
(DOC)

Table S2 **Expression levels of significant (p value >0.05) transcription factors between Cases and Controls. (Mean±SD).**
(DOC)

Table S3 **Genomatix output for 34 TFs showing the frequency of binding sites for each TF on the promoters of different biomarkers from 7 different pathways.**
(DOC)
