# Supplementary material for: Integrative Bioinformatics Analysis of Genomic and Proteomic Approaches to Understand the Transcriptional Regulatory Program in Coronary Artery Disease Pathways
Source: PLoS One. 2013 Feb 28;8(2):e57193. doi: 10.1371/journal.pone.0057193 (PMC3585295; doi:10.1371/journal.pone.0057193)
Supplement: Table S1 — 55 predicted core transcription factors belonging to 23 families. (DOC) [file pone.0057193.s001.doc]

**Table S1**

| 1 | E4BP4 | cAMP-responsive element binding proteins |
| --- | --- | --- |
|  | TAXCREB | cAMP-responsive element binding proteins |
| 2 | NFY | CCAAT binding factors |
|  | CEBPA | Ccaat/Enhancer Binding Protein |
|  | CEBP | Ccaat/Enhancer Binding Protein |
| 3 | E2F4 | E2F-myc activator/cell cycle regulator |
|  | E2F2 | E2F-myc activator/cell cycle regulator |
| 4 | ATF6 | E-box binding factors |
|  | USF | E-box binding factors |
|  | MYCMAX | E-box binding factors |
| 5 | CKROX | EGR/nerve growth factor induced protein C & related factors |
|  | EGR1 | EGR/nerve growth factor induced protein C & related factors |
|  | WT1 | EGR/nerve growth factor induced protein C & related factors |
| 6 | ER | Estrogen response elements |
|  | ESRRA | Estrogen response elements |
| 7 | FHXB | Fork head domain factors |
|  | ILF1 | Fork head domain factors |
| 8 | GATA1 | GATA binding factors |
| 9 | GC | GC-Box factors SP1/GC |
|  | SP1 | GC-Box factors SP1/GC |
|  | SP2 | GC-Box factors SP1/GC |
|  | SP4 | GC-Box factors SP1/GC |
|  | TIEG | GC-Box factors SP1/GC |
| 10 | HOX_PBX | HOX - PBX complexes |
|  | PBX_HOXA9 | HOX - PBX complexes |
| 11 | ETV1 | Human and murine ETS1 factors |
|  | SPI1 | Human and murine ETS1 factors |
|  | ELK1 | Human and murine ETS1 factors |
|  | NRF2 | Human and murine ETS1 factors |
|  | ELF5 | Human and murine ETS1 factors |
| 12 | HRE | Hypoxia inducible factor, bHLH/PAS protein family |
| 13 | BKLF | Krueppel like transcription factors |
|  | KKLF | Krueppel like transcription factors |
|  | KLF6 | Krueppel like transcription factors |
|  | KLF7 | Krueppel like transcription factors |
|  | EKLF | Krueppel like transcription factors |
|  | GKLF | Krueppel like transcription factors |
| 14 | IR2_NGRE | Negative glucocoticoid response elements |
| 15 | NFKAPPAB | Nuclear factor kappa B/c-rel |
|  | NFKAPPAB50 | Nuclear factor kappa B/c-rel |
| 16 | NFAT | Nuclear factor of activated T-cells |
| 17 | P53 | p53 tumor suppressor |
| 18 | PPARG | Peroxisome proliferator-activated receptor |
| 19 | RAR_RXR | RXR heterodimer binding sites |
| 20 | STAT | Signal transducer and activator of transcription |
|  | STAT3 | Signal transducer and activator of transcription |
|  | STAT5 | Signal transducer and activator of transcription |
| 21 | HBP1 | SOX/SRY-sex/testis determinig and related HMG box factors |
|  | HMGA | SOX/SRY-sex/testis determinig and related HMG box factors |
|  | SOX9 | SOX/SRY-sex/testis determinig and related HMG box factors |
|  | SOX30 | SOX/SRY-sex/testis determinig and related HMG box factors |
| 22 | MESP1_2 | Twist subfamily of class B bHLH transcription factors |
|  | HAND2_E12 | Twist subfamily of class B bHLH transcription factors |
|  | TAL1_E2A | Twist subfamily of class B bHLH transcription factors |
| 23 | FTF | Vertebrate steroidogenic factor |
