# Supplementary material for: Integrative Bioinformatics Analysis of Genomic and Proteomic Approaches to Understand the Transcriptional Regulatory Program in Coronary Artery Disease Pathways
Source: PLoS One. 2013 Feb 28;8(2):e57193. doi: 10.1371/journal.pone.0057193 (PMC3585295; doi:10.1371/journal.pone.0057193)
Supplement: Table S2 — Expression levels of significant (p value >0.05) transcription factors between Cases and Controls. (Mean±SD). (DOC) [file pone.0057193.s002.doc]

**Table S2**

| TF | Controls | Cases |
| --- | --- | --- |
| AHR | 10.36±0.43 | 10.79±0.44 |
| AIRE | 7.6±0.24 | 7.25±0.37 |
| ASCL2 | 11.62±0.57 | 10.86±0.53 |
| BACH1 | 5.6±0.59 | 6.23±0.27 |
| CEBPB | 15.93±0.53 | 16.65±0.5 |
| EGR1 | 9.59±2.15 | 12.11±1.36 |
| EGR2 | 7.13±1.78 | 9.78±1.39 |
| EGR3 | 2.76±1.25 | 8.86±1.31 |
| ELF2 | 7.97±0.26 | 7.6±0.18 |
| ELF3 | 3.74±0.74 | 5.15±0.4 |
| EN1 | 3.27±0.78 | 2.62±0.38 |
| ESRRA | 12.44±0.22 | 12.04±0.32 |
| ETS1 | 9.67±0.78 | 10.76±0.91 |
| ETV1 | 2.97±0.75 | 2.27±0.71 |
| GATA5 | 1.74±0.08 | 1.91±0.22 |
| KLF7 | 7.93±0.59 | 8.89±0.33 |
| LMX1B | 7.69±0.75 | 7.02±0.34 |
| MAFB | 12.6±0.66 | 13.31±0.64 |
| MYT1L | 2.03±0.34 | 2.68±0.57 |
| NFE2 | 15.37±0.47 | 14.74±0.7 |
| PAX2 | 4.93±0.45 | 4.14±0.8 |
| PLAGL1 | 6.59±0.31 | 6.87±0.29 |
| PPARG | 3.16±1 | 4.4±1.05 |
| PREB | 11.12±0.21 | 10.84±0.3 |
| RAX | 4.45±0.41 | 4.1±0.23 |
| RFX3 | 6.31±0.42 | 7.08±0.38 |
| SATB1 | 11.93±0.34 | 12.3±0.35 |
| SF1 | 13.02±0.24 | 13.45±0.26 |
| SIP1 | 5.21±0.63 | 4.18±0.93 |
| SOX11 | 1.73±0.13 | 1.95±0.24 |
| SOX4 | 6.21±0.49 | 6.96±0.63 |
| SRF | 7.21±0.71 | 8.46±0.7 |
| TCF7L2 | 5.07±0.91 | 6.08±0.96 |
| ZIC2 | 1.64±0.04 | 1.76±0.11 |
